# Oxidative Stress and the Nrf2/PPARγ Axis in the Endometrium: Insights into Female Fertility

**DOI:** 10.3390/cells13131081

**Published:** 2024-06-22

**Authors:** Peter Artimovič, Zuzana Badovská, Silvia Toporcerová, Ivana Špaková, Lukáš Smolko, Gabriela Sabolová, Eva Kriváková, Miroslava Rabajdová

**Affiliations:** 1 Department of Medical and Clinical Biochemistry, Faculty of Medicine, Pavol Jozef Šafárik University in Košice, Trieda SNP 1, 040 11 Košice, Slovakia; peter.artimovic@student.upjs.sk (P.A.); ivana.spakova@upjs.sk (I.Š.); lukas.smolko@upjs.sk (L.S.); gabriela.sabolova@student.upjs.sk (G.S.); eva.krivakova@student.upjs.sk (E.K.); miroslava.rabajdova@upjs.sk (M.R.); 2 Department of Gynaecology and Obstetrics, Faculty of Medicine, Pavol Jozef Šafárik University in Košice, Trieda SNP 1, 040 11 Košice, Slovakia; silvia.toporcerova@upjs.sk

**Keywords:** Nrf2, PPARγ, oxidative stress, endometrium, fertility, cellular signaling, female, mini review

## Abstract

Successful pregnancy depends on precise molecular regulation of uterine physiology, especially during the menstrual cycle. Deregulated oxidative stress (OS), often influenced by inflammatory changes but also by environmental factors, represents a constant threat to this delicate balance. Oxidative stress induces a reciprocally regulated nuclear factor erythroid 2-related factor 2/peroxisome proliferator-activated receptor-gamma (Nrf2/PPARγ) pathway. However, increased PPARγ activity appears to be a double-edged sword in endometrial physiology. Activated PPARγ attenuates inflammation and attenuates OS to restore redox homeostasis. However, it also interferes with physiological processes during the menstrual cycle, such as hormonal signaling and angiogenesis. This review provides an elucidation of the molecular mechanisms that support the interplay between PPARγ and OS. Additionally, it offers fresh perspectives on the Nrf2/PPARγ pathway concerning endometrial receptivity and its potential implications for infertility.

## 1. Introduction: Endometrial Cycle

The endometrium undergoes a series of morphological and functional changes during the menstrual cycle [1]. The usual cycle length is 28 days, while the highest receptivity for embryo implantation is between the 20th and 24th day, also known as the window of implantation (WOI) [2]. Cyclic changes in ovarian functions divide the menstrual cycle into the follicular phase (0–13 days), the ovulatory phase (14 days), and the luteal phase (15–28 days). Based on the changes in the landscape of the endometrium, the menstrual cycle is divided into three phases: menstrual (days 0–5), proliferative (days 6–13), and finally secretory (days 15–28) [3]. The main regulatory hormones in endometrial physiology are estrogen (E2) and progesterone (P4), whose levels change during the menstrual cycle. Elevated levels of E2 signal the onset of the pre-ovulatory proliferative phase, histologically characterized by the gradual thickening of the endometrium [1,4]. On the molecular level, E2 induces various signaling pathways, mostly kinase cascades or cAMP signaling. Continuing until the ovulatory peak, the effect of E2 is manifested by the increased expression of progesterone receptors (PGR), which enables a smooth transition to the secretory phase after ovulation [5].

In the secretory phase, the endometrium is prepared for decidualization which encompasses all processes involved in the preparation of receptive endometrium, particularly the formation of new tissue termed decidua [3]. Maintaining certain levels of endometrial oxidative stress (OS) is crucial for the transformation of stromal cells into decidual cells [6]. The role of decidualization is to create suitable conditions for blastocyst implantation and embryo growth [2]. Higher expression of antioxidant enzymes in decidual cells protects the embryo from the effects of OS generated during the implantation process [7]. A steep rise in P4 levels induces similar pathways to E2 signaling although they are differentially regulated [8]. If implantation of the blastocyst in the secretory phase fails, menstruation occurs [2]. This occurs due to a significant decrease in P4 levels after the absorption of the corpus luteum, to which the blood vessels react by constricting, resulting in ischemia of the epithelium and its shedding [9]. This process is characterized by the activation of cell death pathways, high levels of OS, and intensive migration of immune cells to the site of damage [10]. At the end of the menstrual phase, the functional layer of the endometrium is regenerated by activated repair mechanisms and stimulated migration of progenitor cells from the basal layer of the endometrium, following the gradual repopulation of the endometrial epithelium [11].

## 2. The Role of Oxidative Stress in the Endometrium

**Figure 1:**
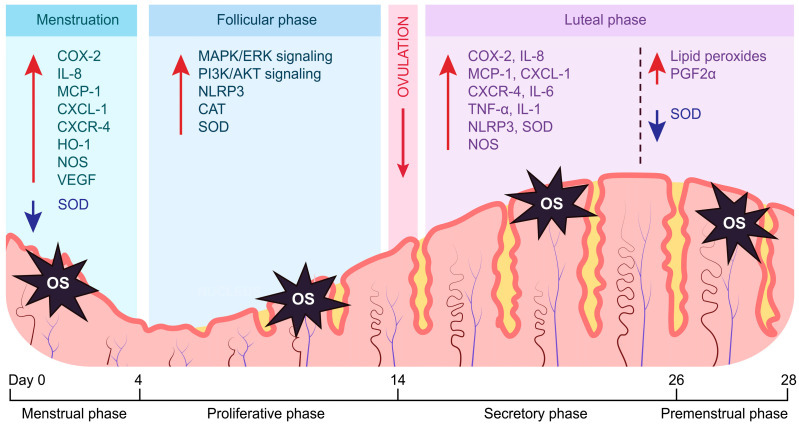
Cyclical changes in the expression of important mediators of endometrial functions induced by OS. Endometrial OS regulates the activity of various inflammatory and angiogenic processes, in addition to directly stimulating the antioxidant response. The follicular phase is characterized by enhanced proliferation pathways (PI3K/AKT—the phosphatidylinositol 3-kinase/protein kinase B, MAPK/ERK—the mitogen-activated protein kinase/extracellular regulated protein kinases), inflammatory processes (NLRP3—NLR family pyrin domain containing 3), and an antioxidant response (CAT—catalase, SOD—superoxide dismutase). A surge in OS during inflammatory cytokine signaling is attenuated by upregulated SOD during the luteal phase, promoting decidualization. The decreased expression of SOD during the transition into the menstrual phase is necessary for the onset of endometrial shedding and subsequent induction of angiogenesis. The differences in the expression of antioxidant genes throughout the endometrial cycle highlight the importance of maintaining optimal OS levels to ensure the precise temporal distribution of phases.

OS participates in numerous physiological functions related to female fertility, encompassing folliculogenesis, oocyte maturation, hormone signaling, and cyclical endometrial changes when maintained at appropriate levels [12]. The uterine endometrium has a unique requirement for oxygen, making OS a significant factor [13]. Its primary role lies in the regulation of endometrial responses to E2 and P4 levels, thus coordinating morphological changes at the molecular level [7]. Approximately 2% of the total oxygen utilized in the generation of energy is redirected to produce reactive oxygen species (ROS) in mitochondrial complexes I and III [14]. The three primary forms of ROS include superoxide anion (O_2_^•−^), hydroxyl (•OH), and hydrogen peroxide (H_2_O_2_), with its ability to permeate biological membranes and transform into the •OH and thus have a toxic effect on cells [15]. Currently, ROS are known modulators of the different phases of the endometrial cycle (Figure 1) [14].

However, if ROS exceed their physiological thresholds, they possess the potential to inflict substantial damage to cellular structures [16]. ROS can also influence the microenvironments associated with follicular and peritoneal fluids, and, as a result, OS can significantly change the expression of genes regulating oocyte quality, activation, implantation, early embryonic development, and other aspects of the female reproductive system, thereby significantly contributing to female infertility [17].

### 2.1. Oxidative Stress Regulates Inflammation in the Endometrium

Inflammation plays both positive and negative roles in female fertility. Physiologically, inflammation is essential for processes like ovulation, menstruation, and implantation [9,18,19]. For instance, controlled inflammatory responses are crucial for the rupture of ovarian follicles during ovulation and for the remodeling of the endometrium during menstruation and implantation [9,19]. However, chronic inflammation, as seen in conditions like endometriosis and polycystic ovary syndrome (PCOS), can negatively impact fertility by causing anatomical distortions and ovulatory dysfunctions [19,20]. Interestingly, dietary interventions that reduce inflammation have been shown to improve fertility outcomes, suggesting the potential for anti-inflammatory diets to support reproductive health [21]. Furthermore, the inflammatory response helps regulate gonadotropin-releasing hormone (GnRH) neurons, essential for reproductive function, indicating a complex but vital role of inflammation in female fertility [22].

Research on the role of OS in endometrial physiology strongly implicates the link between persistent inflammation and oxidative stress [20]. The expression of genes associated with promoting inflammation is attributed to the heightened ROS levels, resulting in ROS-induced inflammatory response [23]. In particular, high levels of ROS have a crucial function in modulating the activity of the transcription factor nuclear factor kappa B (NF-κB) responsible for initiating the activation of various genes encoding proinflammatory cytokines, growth and angiogenic factors, adhesion molecules, as well as inducible enzymes such as nitric oxide synthase (NOS) and cyclooxygenase 2 (COX-2), which react to pathological OS through inflammatory response [13]. Nitric oxide (NO) produced by NOS holds a critical role in normal reproductive processes including oocyte maturation, vascular changes and extracellular matrix (ECM) remodeling during the ovulatory phase, implantation, embryonic development, placental–uterine blood perfusion and opening of the pelvis during birth [24]. However, elevated levels of NO have been associated with embryotoxicity and the inhibition of implantation [25]. Research using the follicular fluid of patients undergoing controlled ovarian stimulation during in vitro fertilization has shown that increased NO levels in follicular fluid are linked to diminished embryo quality and cleavage rates [26]. Furthermore, elevated blood NO concentrations have been observed in infertile women with tubal or peritoneal factor infertility [27]. When follicular fluid NO reaches pathological concentration, it can result in unsuccessful implantation, leading to lower pregnancy rates. In vitro human embryo studies have also suggested that NO may trigger uncontrolled apoptosis and fragmentation in embryonic cells [27,28].

As already mentioned, ROS induces the expression of COX-2 responsible for synthesizing prostaglandins from arachidonic acid [15]. In addition to NF-κB, transcription factor homeobox A10 (HOXA10), induced by higher P4 levels, is also responsible for increased COX-2 transcription, as shown in the mouse model [3,29]. The adhesion phase of implantation is notably modulated by the inflammatory marker COX-2 [30]. This process increases vascular permeability and supports uterine lining attachment, as was seen in LIF-deficient mice [31]. COX-2 expression varies based on embryonic stage and location, primarily found in luminal epithelial and stromal cells, as well as around the implanted blastocyst in pregnant and pseudopregnant rats [32]. Reduced COX-2 expression is associated with implantation and decidualization failures of human decidua, emphasizing its significance [33]. Physiologically increased COX-2 activity leads to the induction of the expression of chemokines such as interleukin 8 (IL-8), membrane cofactor protein 1 (MCP-1), C-X-C chemokine ligand 1 (CXCL-1), and C-X-C chemokine receptor 4 (CXCR-4), which are an essential part of the cross-talk between the endometrium and the blastocyst, mediating the attachment of embryo to uterine lining [18,34]. Pro-inflammatory and chemotactic cytokines are also necessary for the recruitment and activation of phagocytic cells, which are the main producers of ROS and reactive nitrogen species (RNS) involved in blastocyst implantation [14,35,36,37].

Research has shown that ROS play a role in the activation of the NOD-like receptor family, NLRP3 inflammasome, which functions as a crucial intracellular pattern recognition receptor [38]. When NLRP3 combines with the apoptosis-associated speck-like protein containing a CARD (ASC) and cysteine aspartic acid-specific protease-1 (Caspase-1), it forms a canonical inflammasome [39]. Formed NLRP3 complex catalyzes the autocleavage of the Caspase-1 precursor, thereby enhancing the maturation and subsequent release of interleukin-1β (IL-1β) and interleukin-18 (IL-18), initiating and propagating the inflammatory response [40]. However, the physiological functions of NLRP3 in the endometrium are still unclear. NLRP3 exhibits elevated expression levels in the mid-proliferative and mid-secretory phases of the human endometrium and undergoes an increase in transcriptional activity induced by E2 through the estrogen receptor β (ESR2/ERβ) [41,42]. Furthermore, NLRP3 plays a role in fostering embryo implantation and augmenting the epithelial–mesenchymal transition (EMT) of Ishikawa cells through pathways involving both inflammasome-dependent and inflammasome-independent mechanisms [42].

### 2.2. Oxidative Stress Regulates Angiogenesis in the Endometrium

Angiogenesis plays a crucial role in female fertility, impacting processes such as folliculogenesis, ovulation, corpus luteum function, and endometrial changes during menstruation and implantation [9,43]. Dysregulated angiogenesis is linked to reproductive disorders like PCOS, contributing to increased ovarian stromal vascularity and associated infertility [44]. Additionally, aberrant angiogenesis in uterine fibroids is associated with abnormal uterine bleeding and infertility, highlighting potential therapeutic targets [45]. Genetic polymorphisms in angiogenesis-related genes further contribute to recurrent implantation failure in infertile women [46]. Normal endometrial growth during the menstrual cycle is necessary for successful embryo implantation and requires proper regulation of angiogenesis—the process of creating new blood vessels from existing capillaries [2,3]. Endothelial cells forming the capillary bed are influenced by factors produced by the surrounding tissue as well as angiogenic factors circulating in the blood during the menstrual cycle [9]. In the endometrium, angiogenesis is induced by local hypoxia and estrogen receptor signaling predominantly during the follicular phase of the cycle [1,45]. While hypoxia plays a key role in the regulation of endometrial shedding during menstruation, E2 plays a significant role in the reconstruction of new vessels and their rapid growth in the proliferative phase [4,47]. Vascular maturity is achieved during the secretory phase under the influence of P4 [48].

Angiogenesis in the endometrium is modulated by various angiogenic factors of which vascular endothelial growth factor (VEGF) and angiopoietins 1 and 2 (ANG-1/2) are the most important ones [43]. VEGF plays an important role in the regulation of angiogenesis in humans through the activation of two specific tyrosine kinase receptors—VEGFR-1 and VEGFR-2 [49]. A significant part of the biological effects of VEGF is mediated through the VEGFR-2 receptor [50]. Upon binding of VEGF to the receptor, the PI3K/AKT pathway is activated and inhibits apoptosis to promote cell survival and proliferation of endometrial cells [51]. Simultaneous activation of ERK and NF-κB signaling by the PI3K/AKT pathway significantly supports the effect of VEGF [52]. Additionally, stimulation of the PI3K/AKT pathway is associated with NOS expression and elevated OS [53]. Expression of VEGF is also induced by hypoxia through the activation of hypoxia-inducible factor 1α (HIF-1α) [47]. Similarly, ANG-1/2 regulates angiogenesis after binding to their receptor tyrosine kinase with immunoglobulin and epidermal growth factor homology domain 2 (Tie-2). Activation of the ANG-1/Tie-2 pathway results in the stabilization of vasculature by adhesion of pericytes to endothelial cells, while ANG-2 promotes vascular destabilization necessary for VEGF-induced neovascularization [54]. Mechanistically, the ANG-1/Tie-2 pathway activates PI3K/AKT and ERK signaling just like the VEGF/VEGFR pathway [55].

Controlled levels of ROS have also been associated with angiogenic activity by supporting VEGF signaling within the endometrium, contributing to its regenerative processes in each menstrual cycle [56]. The binding of E2 to estrogen receptor 1 (ESR1/ERα) during the proliferative phase is often linked with two well-known pathways: PI3K/AKT pathway and MAPK/ERK pathway [5]. Increased H_2_O_2_ inactivates protein tyrosine phosphatase 1B, which inhibits dephosphorylation of endothelial growth factor receptor (EGFR), thus, also promoting downstream PI3K/AKT and MAPK/ERK pathways [57]. These pathways play a role in enhancing cell survival, growth, inflammation, angiogenesis, metabolic processes, and the absorption of nutrients that are necessary for embryo implantation [58].

Changes in ROS levels during menstrual cycle affect endometrial vasculature [9]. Mid-secretory phase is associated with peak levels of SOD involved in the mitigation of the inflammatory environment accompanying implantation [59]. During the late secretory phase, there is a distinctive rise in lipid peroxide levels and a concurrent decrease in the SOD levels, which signify the transition into the menstrual phase of the endometrial cycle [14]. Reduced levels of estrogen and progesterone result in low SOD expression, thereby giving rise to OS within the uterus. ROS activation has been found to stimulate the secretion of prostaglandin F2α (PGF2α) via the activation of NF-κB [60]. PGF2α produced by COX-2 can induce myometrial contractions, which lead to spiral artery vasoconstriction and epithelial ischemia [61]. Additionally, ROS-mediated NF-κB activation leads to the expression of MCP-1, interleukin 6 (IL-6), tumor necrosis factor α (TNF-α), and interleukin 1 (IL-1), which stimulate neutrophil migration into the stroma [1]. The main function of neutrophils in the stroma is the production of matrix metalloproteinases (MMPs), which promote epithelial desquamation [10]. An important activator of MMP in the menstrual phase is the left–right determining factor 2 (LEFTY-2), a member of the transforming growth factor β (TGF-β) family associated with endometrial bleeding [62]. Its expression is strictly repressed by the influence of P4 to prevent uncontrolled ECM degradation during the secretory phase [3,63]. An uncontrolled rise in OS results in premature endometrial shedding and hinders implantation [64].

Additionally, elevated OS greatly contributes to endothelial cell dysfunction in the uterus which potentially promotes the manifestation of conditions such as pre-eclampsia and endometriosis, which are known as infertility factors [17]. Diverse etiological factors contribute to the onset of endothelial cell dysfunction [65]. Notably, TNF-α has been demonstrated to vastly damage endothelial cells [66]. Under physiological conditions, ROS are quenched by antioxidant systems to prevent the accumulation of harmful molecular factors and thus preserve tissue integrity [67]. The main enzyme of antioxidant defense is manganese superoxide dismutase (Mn-SOD/SOD2), which functions as a compensatory mechanism, countering the negative impact of superoxide anions generated by TNF-α [14]. Endothelial dysfunction can be associated with the generation of ROS via the electron transport chain within the mitochondria, called mitochondrial ROS (mtROS) and NADPH oxidase-ROS (NOX-ROS), that have been observed to mutually influence each other, creating a positive feedback loop [39,68]. Produced ROS might target the NO that plays a pivotal role in regulating endothelial function [28]. As a result, during pathological levels of OS, NO can undergo oxidation and transform into a potent oxidizing agent, peroxynitrite (ONOO−), triggering further oxidative reactions and cellular damage [24]. Active NO signaling may also initiate apoptosis in the process of physiological endometrial shedding [69].

## 3. Antioxidant Defense and the Nrf2 Pathway

The level of OS significantly impacts crucial processes in the endometrium related to endometrial receptivity, embryo implantation, and successful pregnancy, as was mentioned in the previous section [7,14,17]. Their optimal levels are strictly regulated by antioxidant systems [70]. In humans, we differentiate between two distinct antioxidant systems: the enzymatic antioxidant system and the non-enzymatic antioxidant system [71]. The nuclear factor erythroid 2-related factor 2/Kelch ECH-associated protein 1 (Nrf2/KEAP1) pathway represents a main regulator of enzymatic cellular defense against oxidative stress. This response encompasses the expression of vital antioxidant enzymes such as SOD, CAT, glutathione peroxidase (GPx), and heme oxygenase 1 (HO-1) [72]. SOD is a key antioxidant enzyme, which represents the first line of defense against oxidative damage by the dismutation, a simultaneous oxidation and reduction process and decomposition of superoxide radicals into H_2_O_2_ and molecular oxygen [73]. There are three known forms of SOD: the Cu/Zn-SOD (SOD1) and Mn-SOD (SOD2) present in cytosol and mitochondrial and extracellular Fe-SOD (SOD3) [74]. During menstruation, levels of OS may be elevated due to tissue damage and inflammation associated with shedding the endometrial functional layer and its subsequent renewal [10]. SOD activity in the endometrium was shown to increase from the early proliferative phase to the mid-late proliferative phase due to higher levels of E2 and inflammation-mediated ROS production [75]. CAT, another important enzyme of redox signaling, is primarily located within peroxisomes, alongside mitochondria and the nucleus [76]. The primary function of CAT is to facilitate the decomposition of H_2_O_2_ into molecular oxygen and water [77]. In addition, the degradation of H_2_O_2_ is also managed by GPx, which oxidizes glutathione (GSH) as a cofactor [77]. The pool of reduced GSH for this reaction is replenished by glutathione reductase (GR) [78]. HO-1 is an enzyme catalyzing the degradation of heme to biliverdin, CO_2_ and iron [79]. Biliverdin is subsequently converted through the action of biliverdin reductase (BVR) into bilirubin, serving as a scavenger of ROS and providing protection against oxidative damage [80]. A substantial amount of heme is released from endometrial cells during shedding, which increases OS in endometrium due to the redox properties of iron in the heme molecule [81]. Activation of the inflammatory response by OS enhances leukocyte transmigration to degrade heme using HO-1 [82].

**Figure 2:**
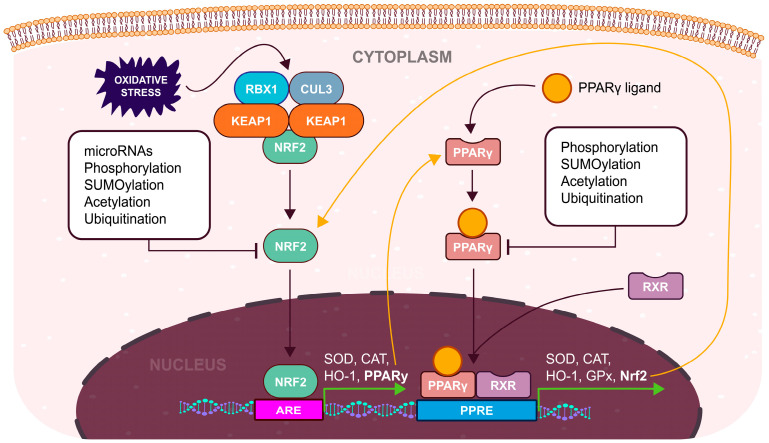
Increased expression of antioxidant genes by activated Nrf2 and PPARγ during OS. Both Nrf2 and PPARγ act as important regulators of the antioxidant response. Elevated OS induces the Nrf2 pathway, stimulating the expression of antioxidant enzymes, along with an increased expression of PPARγ. Reciprocally, activated PPARγ functions like Nrf2, additionally upregulating its expression. This positive feedback loop shown by yellow arrows may be regulated through various mechanisms, such as post-translational modifications or microRNA inhibition. Dysregulation of these pathways has a substantial impact on the management of endometrial OS, contributing to decreased or diminished endometrial receptivity.

Under physiological conditions, Nrf2 is typically complex with its repressor, KEAP1. This complex, in turn, associates with CULLIN 3 (CUL3) and RING-box protein 1 (RBX1), forming the KEAP1/CUL3/RBX1 E3-ubiquitin ligase complex, which facilitates the proteasomal degradation of Nrf2 [83,84]. However, when exposed to oxidative stimuli, ROS interacts with specific cysteine residues on KEAP1, inducing a structural alteration. Consequently, this modification prevents the ubiquitination of Nrf2, enabling its translocation into the nucleus, where it can bind to antioxidant response elements (ARE) regions situated in the promoter regions of antioxidant genes, thereby promoting their transcription (Figure 2) [77]. On the other hand, the non-enzymatic antioxidant system is composed of organic molecules, such as vitamin C, vitamin E, GSH, carotenoids, and melatonin, and trace elements, like copper, zinc, and selenium [70].

In addition to the KEAP1-dependent mechanism, Nrf2 regulation has been shown to occur through various KEAP1-independent pathways. These encompass the transcriptional activation of the Nrf2 gene via the aryl hydrocarbon receptor (AHR) and its nuclear translocator (ARNT), binding to the xenobiotic response element (XRE) [85], post-transcriptional control of *Nrf2* mRNA involving host microRNAs [86,87], post-translational modifications of the Nrf2 protein, including phosphorylation, acetylation [88], and SUMOylation [89], as well as the association of Nrf2 protein with different binding partners [90,91]. This intricate system of transcriptional and post-translational controls on Nrf2 is precisely regulated to adapt its antioxidant functions to shifts in cellular redox balance and maintain homeostasis [92].

## 4. Biochemistry of PPARγ

Apart from the Nrf2, peroxisome proliferator-activated receptor-gamma (PPARγ; NR1C3) serves as a nuclear transcription factor for antioxidant response (Figure 2). Belonging to the nuclear hormone receptor superfamily, PPARs share characteristic functional domains with other superfamily members [93]. These receptors can bind a wide range of ligands, which exhibit diverse actions as agonists, antagonists, or inverse agonists [94]. Initially, PPARs were identified as genotoxic agents that induced the proliferation of peroxisomes in rats [95]. Later, it was discovered that their peroxisome proliferative activity was linked to oxidative DNA damage, primarily caused by the release of H_2_O_2_ from peroxisomes [96].

In humans, three distinct subtypes, PPARα, β/δ, and γ, are encoded by separate genes, each with its unique tissue-specific expression patterns [97]. Notably, the PPARγ gene generates two protein isoforms, namely PPARγ-1 (translated from splice variants γ1-4) and PPARγ-2 (translated from splice variant γ2), which seem to display isoform-specific gene regulation [98,99]. The PPAR-γ1 protein is distributed in a wide range of tissues, including skeletal muscle, liver, colon, cardiac tissue, adipose tissue, immune cells, and various epithelial cell types. On the other hand, PPARγ-2 is mostly located in adipose tissue [100]. Furthermore, depending on its expression in specific cell types or tissues, PPARγ significantly influences a wide array of cellular functions, including proliferation, apoptosis, and differentiation [101]. Additionally, it plays a key role in processes related to inflammation [97], angiogenesis [102], and immune responses [103].

In response to ligand binding, PPARs engage in heterodimerization with the nuclear receptor Retinoid X Receptor (RXR) [104]. This interaction results in a crucial conformational shift, leading to the release of co-repressors and the recruitment of co-activators. Within the regulatory regions of its target genes, a specific DNA-binding sequence is recognized by the PPAR/RXR complex, known as the peroxisome proliferator response element (PPRE) [105]. This element comprises a direct repeat (DR-1) motif, characterized by two half-sites that contain a direct repetition of a hexanucleotide DNA sequence AGGTCA, with a single nucleotide spacer between these repetitions [106,107]. PPREs can be located within various gene regions, including the promoter, introns/exons, or the 3′ downstream region of the target genes [108]. The recruitment of various regulatory protein complexes by the PPAR/RXR heterodimer depends on the specific isoforms of PPARs. This recruitment serves to initiate the transcription of distinct sets of target genes regulated by PPARs, resulting in a unique physiological signature [109]. PPAR co-activators display a spectrum of intrinsic biological functions. These include histone modification with histone acetylases such as cAMP response element-binding protein (CREB)-binding protein (CBP/p300) and steroid receptor coactivator 1 (SRC-1) [110,111], as well as ATPases like the members of the switch/sucrose non-fermentable (SWI/SNF) complex, involved in the dynamic remodeling of chromatin [112]. Additionally, co-activators include proteins bridging the nuclear receptor and the transcription initiation machinery, such as PPAR binding protein/thyroid receptor-associated protein 220 (PBP/TRAP220) [113]. The most well-known co-activator is peroxisome proliferator-activated receptor gamma co-activator 1 α (PGC-1α), which may work in tandem with other co-activators [114].

PPARs form heterodimers with RXR even in the absence of ligands. In this state, the dimer remains associated with co-repressor complexes, which include nuclear receptor corepressor (NCoR), silencing mediator of retinoid and thyroid hormone receptor (SMRT), or receptor-interacting protein 140 (RIP140) [115,116]. These complexes, either directly or indirectly repress gene transcription by recruiting histone deacetylases (HDACs) [117].

The activity of PPARγ is also modulated by post-translational modifications including phosphorylation, SUMOylation, ubiquitination, and acetylation [118]. The phosphorylation of serine 112 mostly represses PPARγ activity [107]. Phosphorylation is mediated either by activated MAPKs or cyclin-dependent kinases (CDKs). In contrast to MAPKs, phosphorylation of serine 112 by CDK7 and 9 enhances its activity which highlights the role of different kinases in the regulation of PPARγ activity [119]. In PPARγ, multiple potential SUMOylation sites, particularly lysine 107, have been extensively studied [118]. Observations indicate an interplay between post-translational modifications, with phosphorylation at serine 112 favoring SUMOylation at lysine 107 [120]. Polyubiquitination of PPARγ usually results in its degradation by the proteasome, therefore an overall reduction in its activity [121]. However, PPARγ can undergo ubiquitination leading to either its degradation or increased protein stability, depending on the site of ubiquitination. Tripartite motif protein 23 (TRIM23) facilitates polyubiquitination, enhancing PPARγ stability [122]. Conversely, F-box only protein 9 (FBXO9) [123] and makorin ring finger protein 1 (MKRN1) have been identified as specific E3 ligases for PPARγ in adipocytes, resulting in ubiquitination and subsequent proteasome-dependent degradation of PPARγ [124].

## 5. Impact of Nrf2/PPARγ Pathway on the Endometrium

Multiple studies have supported the idea of reciprocal regulation between Nrf2 and PPARγ pathways, reinforcing the expression of each other [125,126,127]. Currently, it is directly supported by evidence of the presence of AREs in the PPARγ promoter and the identification of a potential PPRE in the Nrf2 promoter region [128]. The location of these response elements suggests a positive feedback loop connecting the Nrf2 and PPARγ pathways, enabling the simultaneous expression of these transcription factors and their target genes. Therefore, the expression of PPARγ is increased under elevated OS [129].

### 5.1. Negative Impact of the PPARγ Pathway on the Endometrium

**Figure 3:**
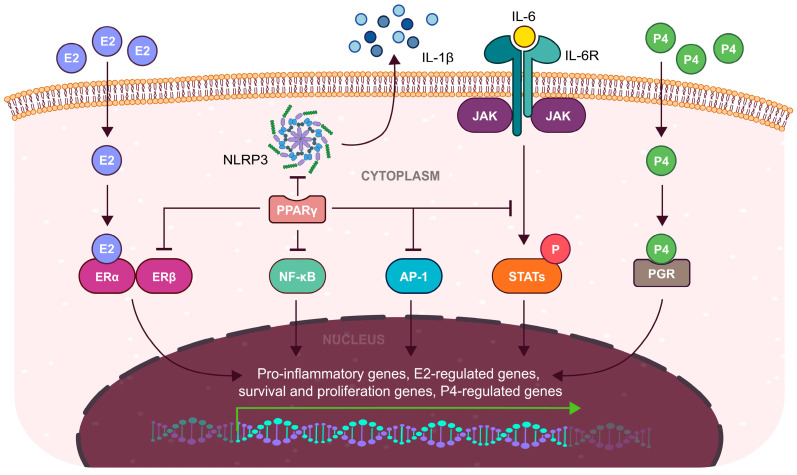
Inhibitory role of PPARγ in endometrial inflammatory processes. Direct interaction between PPARγ and inflammatory transcription factors (NF-κB, AP-1, STAT) has a profound effect on endometrium. Suppression of NF-κB and JAK/STAT/PGR axes results in impaired decidualization. In addition, trans-repression of ERs and AP-1 negatively impacts endometrial thickness, resulting in a loss of endometrial receptivity. Inhibition of NLRP3 inflammasome reduces cytokine levels needed for successful adhesion and implantation of blastocyst. Overall, activated PPARγ represents a potent modulator of endometrial receptivity through interaction with inflammatory molecules under pathological OS. E2—17β-estradiol/estrogen, P4—progesterone.

Increased levels of PPARγ by elevated OS in the endometrium potentially hinder the implantation process of an embryo [18,112,126]. Increased activity of COX-2 is necessary to facilitate the implantation process by inducing the production of cytokines which are important signalization molecules in the blastocyst-endometrium crosstalk [18]. Decreased expression of COX-2 may also negatively impact decidualization by downregulation of VEGF [3,130]. The COX-2 expression mediated by the NF-κB is an important characteristic of the implantation process [1]. However, direct interaction of PPARγ with p65 component of NF-κB results in its inhibition. Additionally, the PPARγ–p65 interaction appears to be modulated by MAPK signaling, as shown by ciglitazone-mediated MAPK phosphorylation of PPARγ, resulting in decreased NF-κB activity [131]. Therefore, inhibitory actions of PPARγ on NF-κB may result in decreased endometrial receptivity and unsuccessful implantation [97]. In addition, estrogen receptor signaling through ERα also modulates the NF-κB activity by a direct association between the two [5]. However, PPARγ interacts with estrogen receptors to interfere with the transcription of their target genes (Figure 3) [132].

As already mentioned, the rise in E2 is the hallmark of the proliferative phase of the endometrial cycle [1]. Inhibition of E2 signaling may result in thinner endometrium, which is associated with lower rates of pregnancy [133]. Interestingly, ERα activates the MAPK pathway which might, in turn, result in the inhibition of NF-κB by phosphorylated PPARγ as observed in HT-29 colon cancer cells [5,131].

Estrogen receptor signaling through the cAMP/protein kinase A (PKA) pathway results in the activation of specificity protein 1 (SP-1) and CREB which induces STAT expression during the proliferative phase [5,134,135]. Adequate levels of STAT5 and STAT3 are necessary to provide functional progesterone receptor signalization during the secretory phase [3,136]. Studies on mice models and breast cancer cells have shown that only phosphorylated STATs can support P4 signalization, while also promoting the expression of prolactin—a biomarker of decidualization [136,137]. Expression of STATs is associated with inflammatory IL-6 signaling but is necessary for the preparation of receptive endometrium [138]. Activation of STATs is mediated by Janus Kinases (JAKs) which are activated by IL-6 or leukemia inhibitory factor (LIF) binding to their receptors [139]. The activation of PPARγ has been shown to inhibit the transcription of STAT5 target genes, signifying a reciprocal negative regulation between PPAR and STAT pathways studied in breast cancer cells [140]. Therefore, PPARγ might inhibit P4 signalization during the secretory phase by trans-repression of STATs which results in the non-receptive endometrium or shift in WOI [137,139,140]. Additionally, PPARγ increases the expression of the suppressor of cytokine signaling (SOCS) protein which negatively regulates JAK/STAT signaling [141].

The importance of NLRP3 inflammasome is in its ability to supply the microenvironment of the endometrium with enough IL-1β to support angiogenesis and remodelation of ECM during embryo implantation [43,142]. However, the activation of NLRP3 inflammasome can be inhibited by PPARγ [143]. Currently, two mechanisms have been proposed to account for PPARγs’ influence on inflammasome activity: the first involves the downregulation of the expression of inflammasome components, while the second mechanism is based on the direct interaction between the DNA-binding domain of PPARγ and NLRP3, which disrupts NLRP3 assembly [143,144]. Studies suggest the third potential mechanism of NLRP3 inhibition, based on the trans-repression of ERβ by PPARγ which may result in the reduction of ERβ-mediated NLRP3 expression [42,145]. However, this mechanism remains to be fully confirmed by experimental data. In this context, the PPARγ/NLRP3 axis may indirectly inhibit EMT which is a necessary step in the implantation of blastocyst, in addition to already known inhibitory pathways of EMT by PPARγ [140,146].

**Figure 4:**
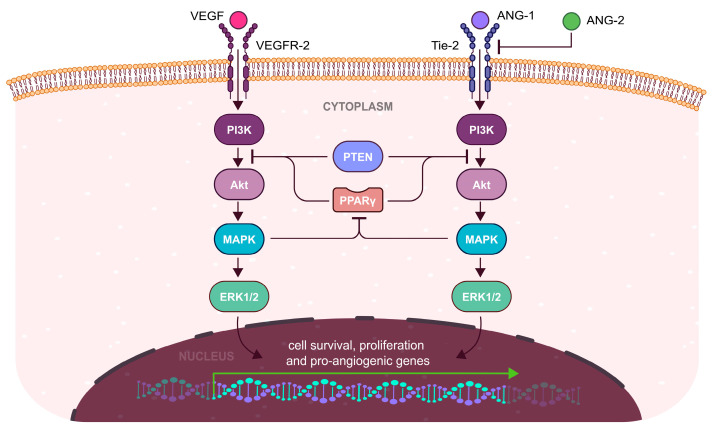
Anti-angiogenic mechanism of PPARγ in the endometrium. Elevated OS and inflammation induce PPARγ activity, resulting in the inhibition of the PI3K/AKT pathway by direct repression of PI3K or upregulation of PTEN—a physiological inhibitor of the PI3K/AKT pathway. This action results in impaired angiogenesis during proliferative and secretory phases, thus negating the effects of estrogen and progesterone signaling on the vasculature. Therefore, endometrial receptivity is reduced due to dysregulated angiogenesis which is necessary for blastocyst implantation and decidualization.

Both inflammation and pathological OS induce the expression of PPARγ as a form of autoregulation [97]. However, activated PPARγ also inhibits angiogenesis by suppressing the PI3K/AKT pathway (Figure 4) similarly to the phosphatase and tensin homolog (PTEN) as was shown in model of bladder cancer chemotherapy [147]. Additionally, the expression of PTEN is directly enhanced by PPARγ binding to PPRE in the promoter region of the gene in human umbilical vein endothelial cells [148]. The presented form of PI3K/AKT pathway regulation has a far-reaching effect on the signalization by important endometrial growth factors, such as fibroblast growth factor (bFGF) or insulin-like growth factor 1 (IGF-1) [149,150]. In the context of female infertility, PPARγ may potentially disrupt the regeneration of endometrium after menstruation through the inhibition of endometrial angiogenesis resulting in the abnormal endometrial functions [3,147].

### 5.2. Positive Impact of the PPARγ Pathway on the Endometrium

Elevated expression of PPARγ positively impacts endometrial functions in two main ways: anti-inflammatory effect and enhanced antioxidant defenses [103,126,151]. PPARγ’s ability to reduce the synthesis of pro-inflammatory mediators in various pathological conditions and experimental models of inflammation is, in part, attributed to its ligand-dependent trans-repression of key transcription factors, notably NF-κB, activation protein 1 (AP-1), and signal transducer and activator of transcription (STAT) (Figure 3) [107,131,140]. Genes containing sites for AP-1 were also found to be upregulated by the activity of ERα, mediated by direct protein–protein interactions [5]. Simultaneous trans-repression of AP-1 and ERα by active PPARγ might contribute to its anti-proliferative and anti-inflammatory properties [140,152]. These interactions are favorable in the context of inflammatory diseases associated with estrogen dominance, such as endometriosis or polycystic ovary syndrome (PCOS). Rising evidence confirms the significant role of PPARγ agonists as a potential treatment for these conditions, especially for women who remain skeptical about standard hormonal therapy [52,153]. Notably, the induction of PPARγ by its synthetic ligands was shown to increase the expression of genes involved in the DNA damage response induced by inflammation-mediated ROS production. Particularly, growth-arrest and DNA-damage inducible 45 beta (GADD45β), CDK1, cyclin A1 (CCNA1), cyclin G1 (CCNG1), and ATM kinase were upregulated in the follicular phase of porcine estrous cycle. These results show another form of cell cycle regulation by PPARγ, supporting its anti-proliferative properties [154]. Additionally, PPARγ exerts an anti-angiogenic effect as was mentioned before, which makes it a potential target for the therapy of gynaecological disorders associated with overactive angiogenesis, such as endometriosis or endometrial cancer [52,151]. The anti-inflammatory effects of PPARγ indirectly support their role as regulators of the cellular response to oxidative stress, although the main antioxidative function of PPARγ primarily stems from the direct activation of antioxidant genes through PPREs located in their promoters [103,155]. Particularly, it promotes the expression of crucial antioxidant enzymes like SOD [156], CAT [157], GPx [158], and HO-1 [159] which maintain optimal OS during the menstrual cycle [13].

Elevated SOD expression during the proliferative phase is mediated by the OS-induced Nrf2/PPARγ pathway [156,160]. Further increase in the mid-secretory phase is associated with decidualization in which a rise in SOD helps to maintain physiological OS through P4 signaling [161]. A decrease in SOD levels in the late secretory phase during the menstrual cycle signifies the initiation of endometrial shedding [162]. Fluctuations in SOD levels correlate with changes in the endometrial OS which might also serve as evidence for fluctuating PPARγ levels during menstrual cycle.

The function of CAT is important in maintaining E2 signaling by stabilizing the expression of estrogen receptors [163]. Increased expression of CAT by PPARγ may serve as a regulatory mechanism for countering PPARγ-mediated inhibition of E2 signaling, thus supporting optimal endometrial functions [132,134,157]. Overactive GPx can rapidly decrease the pool of oxidized GSH. Reduced concentrations of oxidized GSH cause an increase in PPARγ expression directly through the Nrf2 pathway or indirectly through the AHR/ARNT/Nrf2 pathway based on the OS-induced accumulation of toxic metabolites, which may function as ligands for AHR [85].

Additionally, elevated OS induces the Nrf2/PPARγ pathway to enhance the expression of HO-1 [129,159]. This process enables the maintenance of redox homeostasis in the endometrium during shedding, highlighting the importance of PPARγ in the menstrual phase [10,160].

## 6. Conclusions

The dynamic nature of the endometrium makes it highly susceptible to dysregulation. While OS is a natural part of endometrial physiology, its pathological escalation can substantially affect female fertility by reducing endometrial receptivity. In response, endometrial cells protect themselves by activating antioxidant defenses, mainly through the Nrf2 pathway. In addition to upregulating the expression of antioxidant enzymes, the Nrf2 pathway also increases the expression of PPARγ, providing additional support for antioxidant defense. The result is a reduction in and alleviation of inflammation and a reduction in OS levels. The mutual regulation of Nrf2/PPARγ helps in maintaining an optimal endometrial environment for proper blastocyst implantation and decidualization. However, PPARγ can also trans-repress estrogen receptors and suppress PI3K/AKT and JAK/STAT/PGR signaling, which reduces endometrial thickness, destabilizes the vasculature, and impairs decidualization—all key aspects of female fertility. This double-edged nature of PPARγ makes it an interesting target for future therapeutic interventions targeting reproductive disorders related to female infertility. Further research is needed to understand the complex molecular mechanisms behind the role of PPARγ in endometrial physiology, which may lead to new therapeutic options for infertility but also in the process of assisted reproduction.
